# Safety and efficacy of image-guided enzyme-targeting radiosensitization and intraoperative radiotherapy for locally advanced unresectable pancreatic cancer

**DOI:** 10.3892/ol.2014.2101

**Published:** 2014-04-28

**Authors:** AKIHITO NISHIOKA, YASUHIRO OGAWA, KANA MIYATAKE, MICHIKO TADOKORO, MUNENOBU NOGAMI, NORIHIKO HAMADA, KEI KUBOTA, SHINNJI KARIYA, TAKUHIRO KOHSAKI, TOSHIJI SAIBARA, TAKEHIRO OKABAYASHI, KAZUHIRO HANAZAKI

**Affiliations:** 1 Department of Diagnostic Radiology and Radiation Oncology, Kochi University School of Medicine, Kohasu, Okoh-cho, Nankoku-shi, Kochi 783-8505, Japan; 2 Department of First Internal Medicine, Kochi University School of Medicine, Kohasu, Okoh-cho, Nankoku-shi, Kochi 783-8505, Japan; 3 Department of First Surgery, Kochi University School of Medicine, Kohasu, Okoh-cho, Nankoku-shi, Kochi 783-8505, Japan

**Keywords:** pancreatic cancer, radiosensitizer, hydrogen peroxide, image-guided radiotherapy, enzyme-targeting radiosensitization, intraoperative radiotherapy

## Abstract

A novel radiosensitization treatment involving the injection of hydrogen peroxide and sodium hyaluronate, using ultrasonic guidance, into a tumor immediately prior to intraoperative radiotherapy (IORT) was established for patients with stage IVa locally advanced unresectable pancreatic cancer. The aim of the present study was to assess the safety and efficacy of this novel treatment, termed Kochi Oxydol-Radiation Therapy for Unresectable Carcinomas-IORT (KORTUC and IORT). In total, 12 patients were treated with KORTUC-IORT, external-beam radiotherapy and systemic chemotherapy using gemcitabine hydrochloride and S-1. For evaluation of the therapeutic and adverse effects, contrast-enhanced computed tomography was conducted prior to the treatment, and one and six months following KORTUC-IORT. Medical examinations were performed every month at the regularly scheduled follow-up visits. The one- and two-year survival rates were 75 and 25%, respectively, and the median survival time was 16 months. All treatments associated with KORTUC-IORT were well-tolerated by the patients, with a small number of adverse effects and no serious complications. It was identified that the delivery of KORTUC-IORT is safe and effective, in combination with external-beam radiotherapy and systemic chemotherapy, for patients with locally advanced unresectable pancreatic cancer.

## Introduction

**Figure 1:**
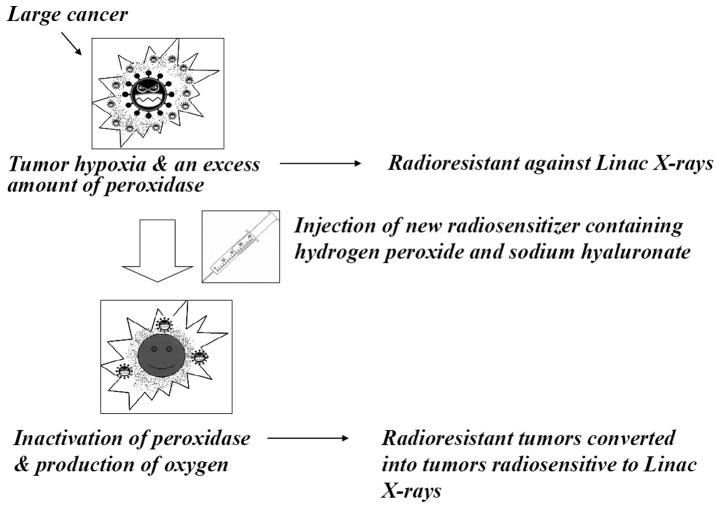
Novel enzyme-targeting radiosensitizing treatment.

In previous experimental studies using the low linear-energy transfer (LET)-radioresistant osteosarcoma cell line, HS-Os-1 (1–3), and the radioresistant prostate cancer cell line, PC-3 (4), it was demonstrated that hydrogen peroxide exhibited strong radiosensitizing effects. Based on these experimental results, a novel radiotherapy technique, Kochi Oxydol-Radiation Therapy for Unresectable Carcinomas, Type-I (KORTUC-I), was developed. It uses hydrogen peroxide and markedly enhances the effects of radiotherapy on various types of superficially exposed and locally advanced neoplasms. Additional radiosensitizing techniques, KORTUC-II and −III, were subsequently developed that used hydrogen peroxide and sodium hyaluronate for intratumoral injection of various types of tumors that are not superficially exposed (Fig. 1). KORTUC-II was approved by the ethics committee of Kochi University (Kochi, Japan) for administration to patients with advanced skin cancers (including malignant melanoma), bone and soft-tissue malignant neoplasms, breast cancer and any type of cancer that had metastasized to the lymph nodes. KORTUC-III was approved for patients with advanced hepatocellular carcinoma. The efficacy of radiotherapy using KORTUC-I and −II has been demonstrated in previous clinical trials (5–8).

Although pancreatic cancer is the 13th most common type of cancer, it is the eigth most common cause of mortality associated with cancer (9). The five-year overall survival rate for pancreatic cancer remains <5% (10). The median survival time of patients with localized, but unresectable disease ranges from eight to 10 months (11). Chemotherapy and external-beam radiotherapy (EBRT) are used as palliative treatments for patients when surgical resection is not feasible. Gemcitabine has been the most commonly used chemotherapeutic agent over the past decade and the benefit of its use, as opposed to fluorouracil, has been demonstrated in patients with advanced pancreatic cancer (12). Numerous studies have shown that intraoperative radiotherapy (IORT) provides a marginal increase in the survival rate for patients with localized resectable pancreatic cancer (13), however, the results were not conclusively in favor for patients with locally advanced unresectable pancreatic cancer (14).

Therefore, a novel radiosensitizer injection technique (KORTUC-IORT) was established for locally advanced unresectable pancreatic cancer, involving the injection of hydrogen peroxide and sodium hyaluronate into the tumor immediately prior to IORT. The aim of the present study was to assess the safety and efficacy of KORTUC-IORT for patients with stage IVa locally advanced unresectable pancreatic cancer.

## Patients and methods

### Patients

A total of 12 patients, five males and seven females, were consecutively enrolled in the KORTUC-IORT trial between February 2008 and September 2010. The ages of the patients ranged from 58 to 79 years with a mean of 69.2 years (Table I). Each patient was enrolled subsequent to providing fully informed, written consent. All patients had surgically confirmed stage IVa locally advanced unresectable pancreatic cancer and were treated with KORTUC-IORT, EBRT and systemic chemotherapy.

### KORTUC-IORT procedure

KORTUC-IORT involved the injection, under ultrasonic guidance, of a maximum of 9 ml radiosensitizer into the tumor tissue immediately prior to the administration of IORT. The radiosensitizer was prepared by adding 0.5 ml of 3% hydrogen peroxide solution (Oxydol; Kenei Pharmaceutical, Co., Ltd., Osaka, Japan) into a commercially available disposable syringe containing 2.5 ml of 1.0% sodium hyaluronate, which is ordinarily used for intra-articular injection in patients with chronic knee joint disorders. Hydrogen peroxide was added immediately prior to use to avoid the degradation of sodium hyaluronate by the oxidative action of hydrogen peroxide. The final concentration was 0.5% hydrogen peroxide and 0.83% sodium hyaluronate.

For IORT, the tumors were irradiated at a dose of 25 Gy in a single fraction with a 12- or 15-MeV electron beam using a linear accelerator (ML-15MDX; Mitsubishi Electric, Co., Ltd., Tokyo, Japan) and no tumor resection was performed. For EBRT, patients received radiation to the abdomen five times per week at a dose of 2 Gy/day in 15 fractions (total dose, 30 Gy) using 10-MV X-rays, prior or subsequent to KORTUC-IORT. Chemotherapy was initiated prior or subsequent to administering KORTUC-IORT and was continued for as long as possible. Chemotherapy consisted of gemcitabine hydrochloride administered intravenously once per week at a dose of 300 mg/body weight during EBRT, and 1000 mg/m^2^ prior or subsequent to radiotherapy with an occasional rest week. S-1, a novel oral anti-cancer agent, is composed of tegafur, gimestat and otastat potassium (15). S-1 was administered orally twice daily at a dose of 80–100 mg/day for 28 days, followed by 14 days without treatment. This cycle was repeated as many times as necessary and Table II summarizes the treatments for each patient.

### Patient follow-up

All patients were examined during regularly scheduled follow-up visits to evaluate the therapeutic and adverse effects of the treatment regimen. Medical and laboratory examinations, including the assessment of the tumor markers carcinoembryonic antigen, cancer antigen 19-9, duke pancreatic monoclonal antigen type 2 and S-pancrease-1 antigen, were performed monthly. Contrast-enhanced computed tomography (CE-CT) was also performed immediately prior and subsequent to KORTUC-IORT at one and six months and every six months thereafter. The therapeutic effects of the treatment regimen were assessed using CE-CT according to the response evaluation criteria in solid tumors (16).

### Statistical analysis

Survival periods were measured starting from the first day of treatment. The Kaplan-Meier method was used to calculate survival analysis.

## Results

### KORTUC-IORT is well tolerated and associated with few side-effects

Treatments associated with KORTUC-IORT were well tolerated in all 12 patients, with few adverse effects and no serious complications. The levels of the serum tumor markers were decreased in 75% of patients (nine of 12 evaluable patients) and in 37.5% of patients (three of eight evaluable patients) at one and six months following KORTUC-IORT, respectively (Table III). The tumor control rate (partial response plus stable disease) was 67% (eight of 12 evaluable patients) and 62.5% (five of eight evaluable patients) at one and six months following KORTUC-IORT, respectively (Table IV).

### Patient follow-up

**Figure 2:**
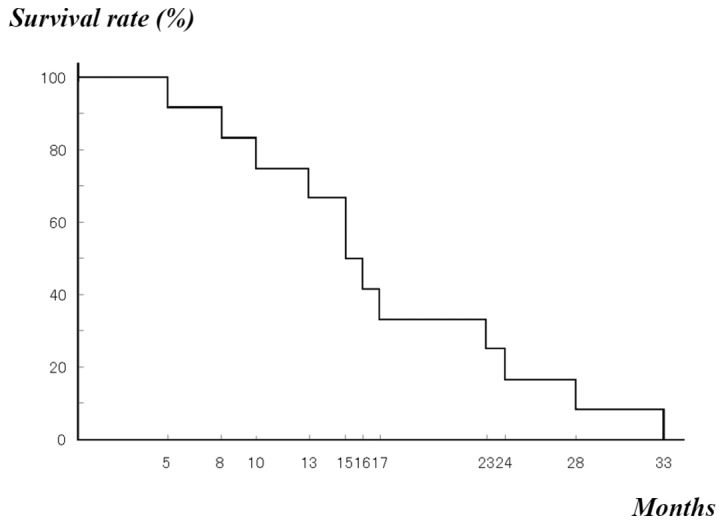
Kaplan-Meier overall survival (n=12).

Follow-up periods for all patients ranged between five and 33 months, and the one- and two-year survival rates were 75 and 25%, respectively. The median survival period was 16 months (Table IV and Fig. 2). There was no association between outcomes, variations in tumor marker concentrations and therapeutic response.

## Discussion

The present study evaluated the safety and efficacy of KORTUC-IORT, which is a novel radiosensitizer that contains hydrogen peroxide and sodium hyaluronate, in patients with stage IVa locally advanced unresectable pancreatic cancer. All treatments associated with KORTUC-IORT were well tolerated by the patients, with a small number of adverse effects and no severe complications.

During the previous 40 years, various radiosensitizers, including metronidazole, misonidazole, etanidazole and nimorazole, have been established to improve the efficacy of radiotherapy (17–19); however these agents, have not been approved for radiosensitization in clinical practice as they are associated with significant side-effects, including peripheral neuropathy. Various radiosensitizing treatments for radioresistant neoplasms have also been established (20–22). However, clinically applicable methods for administration of these agents have not yet been determined. The novel radiosensitizer that was developed in the present study is considered to be particularly safe. The novel radiosensitizer is intended for direct injection into the tumor, and only contains hydrogen peroxide and sodium hyaluronate, which are not generally associated with severe adverse effects as they are degraded into water and oxygen in the presence of peroxidase and catalase. In addition, the solution is directly injected into tumor tissues thereby targeting peroxidase and catalase, which is present in tumor cells. Therefore, this method represents a novel enzyme-targeting radiosensitization treatment with potential indications for various types of low LET-radioresistant neoplasms.

Previous studies have described the treatment of patients with locally advanced pancreatic cancer using combinations of palliative surgery, IORT, EBRT and chemotherapy. In a case series by O’Connor *et al* (23), a median survival time of 12 months was reported. In a retrospective cohort study, Ma *et al* (24) demonstrated a 12.2-month median survival period. In the case series by Furuse *et al* (25), one- and two-year survival rates of 57.9 and 0% were reported, respectively, and a median survival period of 12.9 months. Ogawa *et al* (26) retrospectively analyzed the results of IORT and EBRT performed with or without chemotherapy for patients exhibiting unresectable pancreatic cancer and reported that the two-year overall survival rate and median survival time were 14.7% and 10.5 months, respectively. Using PR-350 as a radiosensitizing treatment, Sunamura *et al* (27) treated locally advanced pancreatic cancer with EBRT and IORT. The study reported a one-year survival rate of 36.4% and a median survival time of 318.5 days. In the present study of 12 patients with stage IVa unresectable pancreatic cancer, the one- and two-year survival rates were 75 and 25%, respectively, and the median survival time was 16 months (Table V). Although the number of patients was small and the follow-up period was short, these results appear to be promising when compared with those that were observed in previous studies. Therefore, the novel radiosensitizing treatment, KORTUC-IORT, is considered to be effective for patients with locally advanced unresectable pancreatic cancer.

In conclusion, KORTUC-IORT may be delivered safely and effectively in combination with current therapies for patients with locally advanced unresectable pancreatic cancer.

## Figures and Tables

**Table I:** Patient characteristics.

Case	Age, years/gender	TNM/stage	Location	Performance status
1	62/F	T4N0M0/IVa	Head	0
2	60/F	T4N0M0/IVa	Body	1
3	71/F	T4N0M0/IVa	Head	1
4	58/F	T4N1M0/IVa	Head	0
5	72/M	T4N0M0/IVa	Body	0
6	68/M	T4N0M0/IVa	Body	1
7	72/F	T4N0M0/IVa	Head	2
8	68/M	T4N0M0/IVa	Body	1
9	75/F	T4N0M0/IVa	Head	1
10	79/F	T4N1M0/IVa	Head	2
11	67/M	T4N0M0/IVa	Head	0
12	78/M	T4N0M0/IVa	Head	0

TNM, tumor-node-metastasis; F, female; M, male.

**Table II:** Unresectable pancreatic cancer treatments.

Case	Treatment
1	S-1 > EBRT > KORTUC-IORT > GEM+S-1
2	EBRT > GEM > KORTUC-IORT > GEM+S-1
3	GEM+S-1 > KORTUC-IORT > EBRT+GEM > GEM
4	GEM+S-1 > KORTUC-IORT > EBRT+GEM > GEM+S-1
5	GEM > KORTUC-IORT > EBRT+GEM > GEM+S-1
6	GEM > KORTUC-IORT > EBRT+GEM > GEM+S-1
7	GEM > KORTUC-IORT > EBRT+GEM > GEM+S-1
8	KORTUC-IORT > EBRT+GEM > GEM+S-1
9	KORTUC-IORT > EBRT+GEM > GEM+S-1
10	KORTUC-IORT > EBRT+GEM > GEM+S-1
11	GEM > KORTUC-IORT > EBRT+GEM > GEM+S-1
12	KORTUC-IORT > EBRT+GEM > GEM+S-1

>, followed by; S-1, tegafur, gimestat and otastat potassium; EBRT, external-beam radiotherapy; KORTUC, Kochi Oxydol-Radiation Therapy for Unresectable Carcinomas; IORT, intraoperative radiotherapy; GEM, gemcitabine hydrochloride.

**Table III:** Adverse effects and tumor markers.

		Effect on serum level of tumor marker	
		
Case	Adverse effect	1 month post treatment	6 months post treatment
1	Mild liver dysfunction	Elevated	Decreased
2	None	Decreased	-
3	None	Elevated	Elevated
4	None	Decreased	Elevated
5	None	Elevated	-
6	None	Decreased	Decreased
7	None	Elevated	-
8	Mild liver dysfunction	Decreased	Elevated
9	Mild liver dysfunction	Decreased	Decreased
10	None	Decreased	-
11	None	Decreased	Decreased
12	Mild liver and renal dysfunction	Decreased	Elevated

-, not evaluable.

**Table IV:** Therapeutic effects and patient outcomes.

Case	Therapeutic effect		Time until patient succumbed, months

	At 1 month	At 6 months	
1	PD	SD	33
2	SD	-	17
3	SD	PD	15
4	SD	PR	24
5	PD	-	28
6	PR	PR	16
7	SD	-	8
8	SD	PD	10
9	PD	SD	13
10	PR	-	5
11	SD	SD	15
12	PD	PD	23

PD, progressive disease; SD, stable disease; PR, partial response; -, not evaluable.

**Table V:** Summary of the principal results of previous studies and the present study.

Author, period (reference)	Number of patients	Treatment	One-year survival rate, %	Two-year survival rate, %	Median survival time
O’Conner *et al*, 1996–2001 (23)	68	Palliat Surg + IORT + EBRT + Chemo	-	-	12 months
Ma *et al*, 1996–2001 (24)	81	Surg + IORT + EBRT + Chemo	-	-	12.2 months
Furuse *et al*, 1995–2001 (25)	30	IORT + EBRT + Chemo	57.9	0	12.9 months
Ogawa *et al*, 1995–2001 (26)	144	IORT + or − EBRT + or − Chemo	-	14.7	10.5 months
Sunamura *et al*, 1999–2002 (27)	48	PR-350 + IORT + EBRT	36.4	-	318.5 days
Present study, 2008–2010	12	KORTUC + IORT + EBRT + Chemo	75.0	25.0	16 months

Palliat, palliative; Surg, surgery; Chemo, chemotherapy; IORT, intraoperative radiotherapy; EBRT, external-beam radiotherapy; PR-350, novel radiosensitizer; KORTUC, Kochi Oxydol-Radiation Therapy for Unresectable Carcinomas; -, no data.
